# StackEPI: identification of cell line-specific enhancer–promoter interactions based on stacking ensemble learning

**DOI:** 10.1186/s12859-022-04821-9

**Published:** 2022-07-11

**Authors:** Yongxian Fan, Binchao Peng

**Affiliations:** School of Computer Science and Information Security, Guilin University of Electronic Technology, Guilin, 541004 China

**Keywords:** Enhancer–promoter interaction, Bioinformatics, Machine learning, Stacking strategy, Feature extraction

## Abstract

**Background:**

Understanding the regulatory role of enhancer–promoter interactions (EPIs) on specific gene expression in cells contributes to the understanding of gene regulation, cell differentiation, etc., and its identification has been a challenging task. On the one hand, using traditional wet experimental methods to identify EPIs often means a lot of human labor and time costs. On the other hand, although the currently proposed computational methods have good recognition effects, they generally require a long training time.

**Results:**

In this study, we studied the EPIs of six human cell lines and designed a cell line-specific EPIs prediction method based on a stacking ensemble learning strategy, which has better prediction performance and faster training speed, called StackEPI. Specifically, by combining different encoding schemes and machine learning methods, our prediction method can extract the cell line-specific effective information of enhancer and promoter gene sequences comprehensively and in many directions, and make accurate recognition of cell line-specific EPIs. Ultimately, the source code to implement StackEPI and experimental data involved in the experiment are available at https://github.com/20032303092/StackEPI.git.

**Conclusions:**

The comparison results show that our model can deliver better performance on the problem of identifying cell line-specific EPIs and outperform other state-of-the-art models. In addition, our model also has a more efficient computation speed.

**Supplementary Information:**

The online version contains supplementary material available at 10.1186/s12859-022-04821-9.

## Background

Enhancer–promoter interactions (EPIs) play a crucial role in the transcriptional regulation of genes and in human disease progression and cell differentiation. A promoter is a short DNA segment with a sequence length varying between 100 and 1000 base pairs, located upstream of a specific gene [1, 2]. Promoters play critical regulatory roles at different stages of gene expression and contain a wealth of information about gene annotation. Enhancers are vital cis-regulatory elements that regulate spatiotemporal gene expression and act on their target genes at a distance [3]. The enhancer sequence is about 50–1500 base pairs, and further activates the transcription level of its target gene through intimate contact with the promoter [4]. The interaction mechanism of enhancer–promoter is very complex. Multiple enhancers can control a promoter, and multiple promoters can also be regulated by a single enhancer. The interaction distances of interacting enhancer and promoter pairs vary significantly due to the three-dimensional folding of chromatin, ranging from thousands to millions of base pairs apart [5–9].

In the past, with the rapid development of high-throughput sequencing technology, wet experimental methods based on chromosome conformation capture (3C) [10] and its variants were mainly used to study EPIs, such as high-throughput chromosome conformation capture (Hi-C) [11] and chromatin interaction analysis by paired-end tag sequencing (ChIA-PET) [1, 12]. However, these experimental methods have the characteristics of severe technical difficulty, long experiment time, and high labor and material cost. Fortunately, these wet experiments provide valuable data that makes it feasible to study EPIs using computational methods.

In recent years, many computational methods using machine learning algorithms to identify EPIs have been proposed. From the perspective of data usage, it can be divided into two categories: one is the prediction method based on the gene sequence, and the other is the prediction method based on the epigenome. For example, Whalen et al. [2] proposed a predictive model TargetFinder in 2016, which predicted EPIs using functional genomics signals of enhancers, promoters and intermediate regions, and later used the benchmark dataset for EPIs by most researchers [13–19] are all derived from this; in 2017, Yang et al. [14] proposed a prediction model PEP using only gene sequence features and combined word embedding method [20]. From the perspective of computational methods, the existing prediction methods for identifying EPIs can be divided into unsupervised and supervised learning. Unsupervised learning methods are divided into correlation-based and decomposition-based methods and supervised learning methods are separated into methods for training classifiers with machine learning and regression-based methods [21]. In addition, with the rise and wide application of neural networks, many EPIs prediction methods of deep learning have been proposed. For example, Mao et al. [13] built a model EPIANN for predicting EPIs based on attention mechanism and position-based feature decoding; Singh et al. [15] combined convolutional neural network (CNN) and long short-term memory (LSTM) in 2018, considering the long-term dependence of DNA sequence, and proposed the method SPEID. In 2019, a method called SIMCNN [16] referred to SPEID and pointed out that EPIs can be accurately predicted with only the CNN structure. In 2020, four methods, EPIVAN [17], EPI-DLMH [19], SEPT [22], and EPPredictor [18], were proposed successively. EPIVAN uses pre-trained DNA2vec vectors for sequence embedding to extract features. EPI-DLMH makes predictions by matching heuristic method. SEPT is based on domain adversarial networks to study EPIs across cell lines. And EPPredictor predicts EPIs using epigenomic and sequence-based features.

**Fig. 1:**
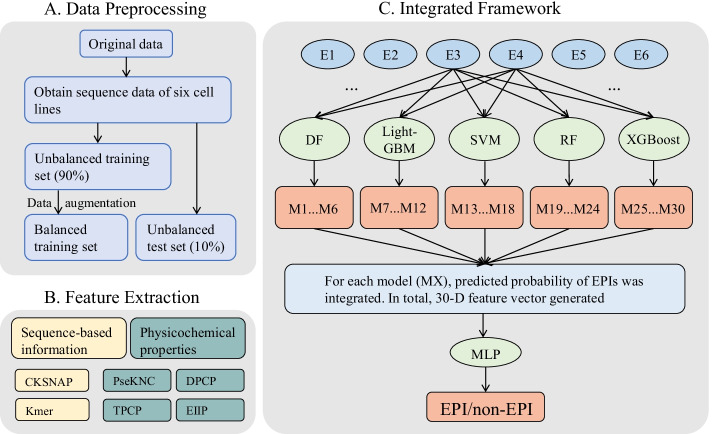
StackEPI overview. It includes **a** data preprocessing, **b** feature extraction, and **c** integrated framework

Different from the above methods, we review, analyze and compare 6 different feature coding methods and 7 commonly used machine learning algorithms, in which Multi-Layer Perceptron (MLP) and Logistic Regression (LR) algorithms are only used as meta-classifiers. Then we propose a novel stacking ensemble framework to identify cell line-specific EPIs named StackEPI. Figure 1 contains the workflow of StackEPI methodology. We find that by using only DNA sequence data and a two-layer stacking strategy to integrate traditional machine learning methods, compared with the most advanced existing methods, our method not only has the advantage of shorter training time in model training speed but also further improves the effect of identifying cell line-specific EPIs.

## Results and discussion

### Pairwise evaluation of 6 feature encodings for 5 machine learning algorithms

**Fig. 2:**
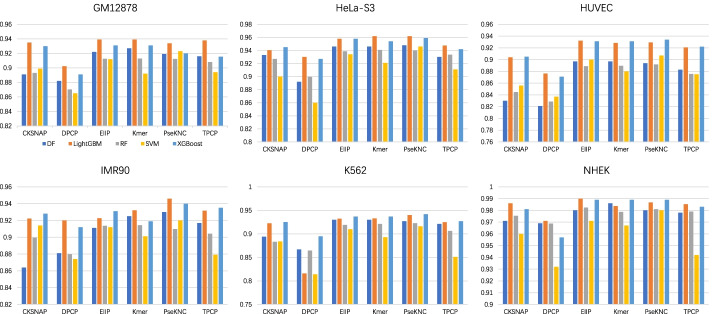
AUROC of the baseline models combined with 6 feature encodings and 5 machine learning algorithms on 6 cell lines

**Fig. 3:**
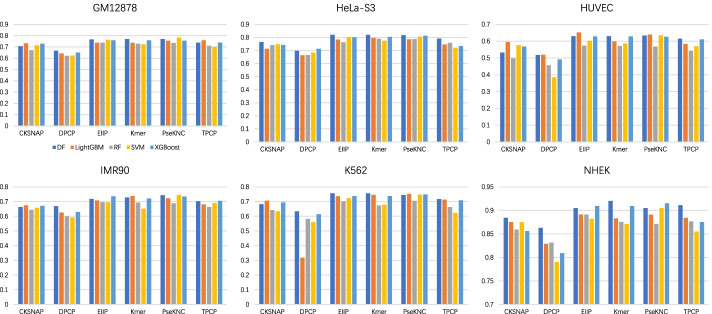
AUPR of the baseline models combined with 6 feature encodings and 5 machine learning algorithms on 6 cell lines

**Fig. 4:**
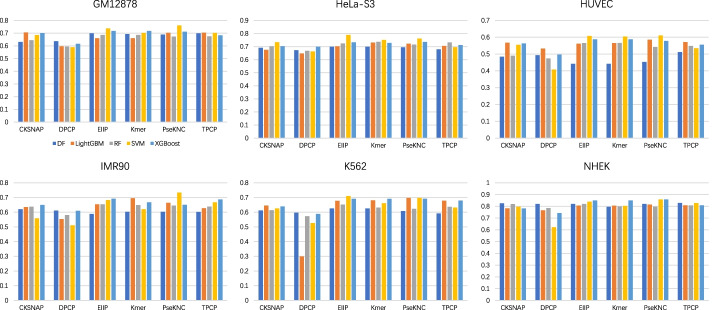
F1-score of the baseline models combined with 6 feature encodings and 5 machine learning algorithms on 6 cell lines

We performed pairwise combination of 6 feature encodings (CKSNAP, Kmer, DPCP, TPCP, EIIP [23], PseKNC [24, 25]) and 5 machine learning algorithms (Deep Forest (DF) [26], Support Vector Machine (SVM), Random Forest (RF) [27], LightGBM [28], XGBoost [29]). Experiments and evaluations were carried out on 6 cell lines (GM12878, HeLa-S3, HUVEC, IMR90, K562, NHEK). Finally, as shown in Figs. 2, 3 and 4, we obtained the experimental results of each cell line in terms of area under receiver operating characteristic curve (AUROC) [30], area under precision-recall curve (AUPR) [31], and F1-score.

First, from the point of view of feature coding, the prediction performance obtained by using the encoding method DPCP is inferior to the other five encoding schemes. Compared with DPCP, TPCP with trinucleotide composition as the main body has a better performance. The three feature encodings of EIIP, Kmer and PseKNC also obtained satisfactory performance depending on different information extraction methods, which indicated that EIIP, Kmer, and PseKNC extracted effective sequence information from the gene sequences of enhancers and promoters. Second, we explored the performance of different machine learning algorithms in terms of AUROC (in Fig. 2). Under the premise of controlling for variables, it is found that LightGBM and XGBoost outperformed the other 3 machine learning algorithms. These two algorithms have good EPI recognition performance in almost all cases, and they are also very efficient to train. Finally, by comparing the cell line-specific prediction results, the baseline models have the best effect on NHEK, with AUROC, AUPR, and F1-score reaching 0.990 (EIIP + LightGBM), 0.920 (Kmer + DF), and 0.859 (PseKNC + XGBoost), respectively. To sum up, all baseline models effectively extract the feature information of gene sequences for EPI recognition, and the performance of these models in each specific cell line is not consistent, which provides multi-level characteristic information for the training of meta-models.

### Ensemble results of candidate meta-classifiers

To determine the most suitable machine learning algorithm as a meta-classifier, we added two classical classifier algorithms, MLP, and LR, in addition to the five algorithms used in the base classifiers. The reason for considering adding these two algorithms as one of the candidate meta-classifiers is that choosing simple computational methods as meta-classifiers in ensemble learning often yields excellent results. Table 1 records the detailed experimental results of 7 different meta-classifiers in 6 cell lines. Notably, we found that an ensemble model with MLP as a meta-classifier, named StackEPI, achieved the best predictions on multiple cell lines given the same feature vector input. StackEPI uses 6 kinds of feature encodings and 5 kinds of calculation methods to form 30 base classifiers, and the obtained predicted probability value is used as the input of the meta-classifier. Especially in the performance of AUROC, StackEPI achieved the best results in 5 (GM12878, HeLa-S3, HUVEC, K562, NHEK) of the 6 cell lines, while the AUROC on IMR90 (93.3%) was 0.7% worse than the best AUROC (94%). In addition, in the comparison of AUPR, StackEPI has achieved performance advantages in 4 cell lines (GM12878, HeLa-S3, HUVEC, K562), which are 0.6%, 0.6%, 1%, 0.6% higher than the second place, respectively. Compared with the optimal AUPR on the other two cell lines (IMR90 and NHEK), the difference was 0.1% and 0.1%, respectively. Finally, the results in Table 1 also show that the ensemble model using LR as the meta-classifier achieves similar results to StackEPI, and slightly outperforms StackEPI on IMR90. In general, StackEPI adopts the stacking ensemble learning method and has a more powerful EPI recognition ability than a single baseline model by integrating feature information from 30 baseline models.

**Table 1:** Detailed performance evaluation of StackEPI's candidate meta-classifiers in 6 cell lines

Cell lines	Meta-models	AUROC	AUPR	F1-score
GM12878	DF	0.925	0.760	0.585
	LightGBM	0.929	0.716	0.672
	RF	0.865	0.708	0.701
	SVM	0.936	0.773	0.705
	XGBoost	0.915	0.722	0.439
	LR	0.932	0.772	**0.722**
	MLP	**0.939**	**0.779**	0.715
HeLa-S3	DF	0.948	0.810	0.665
	LightGBM	0.945	0.783	0.719
	RF	0.898	0.759	0.736
	SVM	0.948	0.816	0.730
	XGBoost	0.893	0.697	0.380
	LR	0.949	0.815	0.746
	MLP	**0.957**	**0.822**	**0.753**
HUVEC	DF	0.902	0.632	0.426
	LightGBM	0.892	0.587	0.382
	RF	0.822	0.544	0.518
	SVM	0.838	0.616	**0.614**
	XGBoost	0.854	0.567	0.577
	LR	0.909	0.641	0.574
	MLP	**0.910**	**0.651**	0.601
IMR90	DF	0.931	0.717	0.568
	LightGBM	0.860	0.625	0.620
	RF	0.849	0.665	0.624
	SVM	0.922	0.629	0.449
	XGBoost	0.882	0.696	0.281
	LR	**0.940**	**0.739**	0.686
	MLP	0.933	0.738	**0.692**
K562	DF	0.924	0.735	0.560
	LightGBM	0.929	0.688	0.449
	RF	0.859	0.677	0.655
	SVM	0.928	0.745	0.662
	XGBoost	0.900	0.697	0.655
	LR	0.927	0.745	0.680
	MLP	**0.930**	**0.748**	**0.717**
NHEK	DF	0.980	0.904	0.780
	LightGBM	0.984	0.892	0.688
	RF	0.953	0.871	0.841
	SVM	0.984	0.913	**0.849**
	XGBoost	0.966	**0.915**	0.681
	LR	**0.985**	0.913	**0.849**
	MLP	**0.985**	0.914	0.844

### StackEPI-faster: faster and stronger predictor of EPIs

In the first two sections, we assessed the performance of 30 baseline models and 7 different meta-models for each cell line, respectively. Combined with the fast and accurate characteristics of the two algorithms, LightGBM and XGBoost, we explored a novel stacking ensemble model, StackEPI-faster, which is expected to achieve faster training speed and higher performance. StackEPI-faster selected four feature encodings (CKSNAP, EIIP, Kmer, and PseKNC) and two statistical methods (LightGBM and XGBoost) to construct 8 base classifiers and selected MLP and LR as the candidate algorithms for the meta-classifier. We first input the enhancer and promoter gene sequences into the 8 baseline models and get 8 predicted probability values. Then we feed these predicted probability values as new features to the MLP classifier or LR classifier. Finally, we get a trained ensemble model and test our model in an independent test set. The final prediction effect of the integrative model on each cell line is shown in Table 2. We can see that the LR classifier is more suitable for StackEPI-faster's meta-classifier than the MLP classifier, and only the experimental results obtained on HUVEC are slightly inferior to MLP. The training time of StackEPI-faster is astonishingly shortened by hundreds of times compared to other prediction methods, which will be described in the training time comparison section.

**Table 2:** Detailed performance evaluation of StackEPI-faster's candidate meta-classifiers in 6 cell lines

Cell lines	Meta-models	AUROC	AUPR	F1-score
GM12878	LR	**0.945**	**0.777**	**0.725**
	MLP	**0.945**	0.775	0.713
HeLa-S3	LR	**0.962**	0.808	**0.737**
	MLP	0.961	**0.810**	0.733
HUVEC	LR	0.935	0.642	0.578
	MLP	**0.937**	**0.644**	**0.601**
IMR90	LR	**0.946**	**0.737**	**0.691**
	MLP	0.944	0.734	0.686
K562	LR	**0.944**	**0.760**	0.684
	MLP	**0.944**	0.758	**0.701**
NHEK	LR	**0.990**	**0.913**	**0.836**
	MLP	0.989	0.912	0.830

### Compared with public methods

To verify the effectiveness and superiority of our prediction method, we compare our method with two state-of-the-art methods, namely EPIVAN and EPI-DLMH. To be fair, first we chose the same benchmark dataset of 6 cell lines and used the identical training set and independent test set. Second, each comparison method follows the basic training procedure referred to in the literature for model training. Finally, all our experiments are conducted on the same machine (Ubuntu 20.04.4 LTS, 3070 GPU). We give the prediction tables of AUROC, AUPR, and F1-score in Tables 3, 4 and 5. Overall, we outperformed EPIVAN and EPI-DLMH in AUROC, outperformed EPIVAN and slightly inferior to EPI-DLMH in AUPR and F1-score as a whole. However, the training time of EPI-DLMH is very expensive, which takes more than ten hours, and our method can control the training time within 5 min (in the Faster training speed section).

**Table 3:** AUROC of EPIVAN, EPI-DLMH, StackEPI(-faster) in 6 cell lines

Methods	GM12878	HeLa-S3	HUVEC	IMR90	K562	NHEK
EPIVAN	0.919	0.954	0.933	0.887	0.937	0.986
EPI-DLMH	0.929	0.962	0.936	0.893	0.939	0.987
StackEPI	0.939	0.957	0.910	0.933	0.930	0.985
StackEPI-faster	0.945	0.962	0.937	0.946	0.944	0.990

**Table 4:** AUPR of EPIVAN, EPI-DLMH, StackEPI(-faster) in 6 cell lines

Methods	GM12878	HeLa-S3	HUVEC	IMR90	K562	NHEK
EPIVAN	0.756	0.819	0.640	0.688	0.752	0.910
EPI-DLMH	0.789	0.863	0.709	0.712	0.767	0.911
StackEPI	0.779	0.822	0.651	0.738	0.748	0.914
StackEPI-faster	0.777	0.810	0.644	0.737	0.760	0.913

**Table 5:** F1-score of EPIVAN, EPI-DLMH, StackEPI(-faster) in 6 cell lines

Methods	GM12878	HeLa-S3	HUVEC	IMR90	K562	NHEK
EPIVAN	0.700	0.717	0.590	0.628	0.678	0.852
EPI-DLMH	0.751	0.809	0.619	0.692	0.712	0.860
StackEPI	0.715	0.753	0.601	0.692	0.717	0.844
StackEPI-faster	0.725	0.737	0.601	0.691	0.701	0.836

Table 3 shows the performance comparison of our model and comparative models on the AUROC metric. The AUROC performance of StackEPI-faster outperformed other methods (EPIVAN, EPI-DLMH) on all cell lines. Specifically, StackEPI-faster has 2.6%, 0.8%, 0.4%, 5.9%, 0.7% and 0.4% higher AUROC on each cell line compared to EPIVAN; compared to EPI-DLMH, the AUROC of our model is 1.6%, 0.1%, 5.3%, 0.5%, and 0.3% higher on the five cell lines GM12878, HUVEC, IMR90, K562, and NHEK, respectively, and remains the same on HeLa-S3. Tables 4 and 5 compare our model with other state-of-the-art models on AUPR and F1-score, respectively. In AUPR comparison, StackEPI-faster surpasses EPIVAN in GM12878, HUVEC, IMR90, K562, and NHEK, by 2.1%, 0.4%, 4.9%, 0.8%, and 0.3%, respectively. On F1-score, StackEPI-faster outperforms EPIVAN by 2.5%, 2%, 1.1%, 6.3%, and 2.3% in GM12878, HeLa-S3, HUVEC, IMR90, and K562, respectively. In summary, our model has different aspects and degrees of superiority over state-of-the-art models on each cell line and outright superiority in terms of AUROC.

### Faster training speed

In addition, in terms of model training time, we further compared the training time of the existing model with that of our proposed method. We found that our approach has unparalleled advantages over other methods. In our experiments, we use the laptop's 3070 GPU to train all models. Table 6 details the training time of EPIVAN, EPI-DLMH, StackEPI, and StackEPI-faster in each cell line. Specifically, EPI-DLMH takes the longest training time, and StackEPI-faster has the shortest training time and a considerable improvement, shortening the time up to hundreds of times. The results demonstrate that our model is more efficient than state-of-the-art models.

**Table 6:** Training duration (h) of EPIVAN, EPI-DLMH, StackEPI(-faster) in 6 cell lines

Methods	GM12878	HeLa-S3	HUVEC	IMR90	K562	NHEK
EPIVAN	1.870	1.299	1.460	1.063	1.664	1.079
EPI-DLMH	16.891	12.304	14.105	10.174	16.041	10.431
StackEPI	1.135	0.807	0.694	0.498	0.983	0.515
StackEPI-faster	0.088	0.046	0.043	0.031	0.060	0.036

## Conclusions

In this study, we propose a method called StackEPI, which combines multiple feature encodings and machine learning algorithms, adopts a stacking ensemble strategy and performs EPI prediction only through enhancer and promoter gene sequences. First, we use 6 feature encodings and 5 machine learning algorithms to construct 30 baseline models based on the benchmark dataset and then obtain a 30-dimensional feature vector generated from all baseline models and composed of predicted probability values. The new vector is input to the meta-classifier to get the final prediction as the ultimate result. In an ensemble manner, our ensemble model synthesizes the advantages of baseline models, which is shown in that our ensemble model has better scores on AUROC, AUPR, and F1-score than the single baseline model. Compared with the most advanced prediction methods, our method has breakneck training speed and better AUROC performance. And theoretically, the input of our approach does not have strict requirements on the length of enhancer and promoter gene sequences. Therefore, it may become a useful tool for fast and accurate prediction of cell line-specific EPIs.

## Methods

### The benchmark dataset

Our experimental data came from TargetFinder and used the same EPI benchmark dataset as EPIVAN. The dataset was provided initially by SPEID, and the complete data can be downloaded from http://genome.compbio.cs.cmu.edu/~sss1/SP-EID/all_sequence_data.h5. The details of the dataset are given in Table 7. The benchmark dataset contains six human cell lines of varying sample sizes: GM12878, HUVEC, HeLa-S3, IMR90, K562, and NHEK. From Table 7, it can be noted that the ratio of positive to negative samples for each cell line is highly imbalanced, with a positive to negative ratio of about 1:20. To address the negative impact of the imbalance between positive and negative samples on model training, we followed the same data processing method as in EPIVAN. To balance the data, they expanded the number of positive samples per cell line to 20 times the original number. While the data were augmented, they also fixed the sequence lengths of enhancers and promoters using 3kbp and 2kbp windows, respectively, to extract more information around the sequences.

**Table 7:** Number of positive and negative samples for 6 cell lines in the original dataset

Cell lines	Positive samples	Negative samples
GM12878	2113	42,200
HeLa-S3	1740	34,800
HUVEC	1524	30,400
IMR90	1254	25,000
K562	1977	39,500
NHEK	1291	25,600
Total	9899	197,500

The six human cell lines, GM12878, HeLa-S3, HUVEC, IMR90, K562 and NHEK, represent lymphoblastoid cells, umbilical vein endothelial cells, ectoderm-lineage cells from a patient with cervical cancer, fetal lung fibroblasts, mesoderm-lineage cells from a patient with leukemia, and epidermal keratinocytes, respectively. Total represents the sum of six cell lines

### Feature encodings

We tried 6 feature encodings commonly used in biological research and analyzed their ability to identify EPIs under different classifiers. The pros and cons of feature encoding schemes are generally positively correlated with model performance [32–34]. The six encoding schemes are (a) sequence-based features, CKSNAP, Kmer; (b) physicochemical properties-based features, DPCP, TPCP, EIIP, and PseKNC.

At present, the physicochemical properties of k-tuple nucleotides are also commonly used in computational prediction of bioinformatics, such as type III secreted effectors [35], type VI secreted effectors [36], enhancers [37], IL-6 inducing peptides [38], and replication origin sites [39]. Recently, Zhang et al. [40] sorted out 182 physicochemical properties from the existing physicochemical properties database and published scientific literature, which are stored in the web-based KNIndex database, which can be obtained on https://knindex.pufengdu.org. In this study, we used the standardized physicochemical properties of k-tuple nucleotides provided by Zhang. In brief, each feature encoding is described as follows.

*CKSNAP* is the composition of *K*-spaced nucleic acid pairs and is widely used in various studies in the bioinformatics field [37, 41, 42]. The feature encoding method calculates the occurrence frequency of nucleotide pairs spaced by arbitrary $k$ nucleotides in a gene sequence, that is, the occurrence frequency of nucleotide pairs composed of two nucleotides at subscript $i$ and subscript $i + k + 1$, respectively. Its calculation can be defined as follows:

1 $$\left( {\frac{{N_{{A{*}A}} }}{{N_{Total} }},\frac{{N_{{A{*}C}} }}{{N_{Total} }},\frac{{N_{{A{*}G}} }}{{N_{Total} }},\frac{{N_{{A{*}T}} }}{{N_{Total} }}, \ldots ,\frac{{N_{{T{*}A}} }}{{N_{Total} }},\frac{{N_{{T{*}C}} }}{{N_{Total} }},\frac{{N_{{T{*}G}} }}{{N_{Total} }},\frac{{N_{{T{*}T}} }}{{N_{Total} }}} \right)_{K = k}$$

where the symbol ${*}$ represents the $k$ nucleotides $S \left( {S \in \left\{ {A,T,G,C} \right\}} \right)$ spaced between nucleotide pairs $XY$, $N_{Total}$ and $N_{X*Y}$ represent the total composition number of $k$-spaced nucleotide pairs in the gene sequence and the number of $k$-spaced nucleotide pairs $XY$, respectively. For example, with $k = 0$, we can obtain a 16-dimensional digital vector consisting of the occurrence frequency of 16 classes of nucleotide pairs (AA, AC, AG, AT, …, TA, TC, TG, TT) with a spacing of 0:

2 $$\left( {\frac{{N_{AA} }}{{N_{Total} }},\frac{{N_{AC} }}{{N_{Total} }},\frac{{N_{AG} }}{{N_{Total} }},\frac{{N_{AT} }}{{N_{Total} }}, \ldots ,\frac{{N_{TA} }}{{N_{Total} }},\frac{{N_{TC} }}{{N_{Total} }},\frac{{N_{TG} }}{{N_{Total} }},\frac{{N_{TT} }}{{N_{Total} }}} \right)_{K = 0}$$

in which

3 $$N_{Total} = N_{AA} + N_{AC} + ... + N_{TG} + N_{TT}$$

In this study, we first set $K = 0, 1, 2, 3, 4,{ }\;{\text{and}} \;5$, then concatenate these obtained vectors horizontally, and finally get a 96-dimensional feature vector. It is worth noting that some sequences in the dataset contain the unknown base 'N'. We excluded the calculation part containing the base 'N' in the process of feature extraction. Extra feature encodings also follow this processing rule and will not be repeated here.

*Kmer* describes the occurrence frequency of $k$ neighboring nucleotides in the gene sequence. We set the step size of traversing the gene sequence to 1, and concatenate the feature vectors generated by nucleotide acid composition (NAC), dinucleotide composition (DNC), trinucleotide composition (TNC), and tetranucleotide composition (TeNC). Their calculations are as follows:

4 $$NAC = \left( {\frac{{N_{A} }}{{N_{total} }},\frac{{N_{C} }}{{N_{total} }},\frac{{N_{G} }}{{N_{total} }},\frac{{N_{T} }}{{N_{total} }}} \right)_{k = 1}$$

5 $$DNC = \left( {\frac{{N_{AA} }}{{N_{total} }},\frac{{N_{AC} }}{{N_{total} }}, \ldots ,\frac{{N_{TG} }}{{N_{total} }},\frac{{N_{TT} }}{{N_{total} }}} \right)_{k = 2}$$

6 $$TNC = \left( {\frac{{N_{AAA} }}{{N_{total} }},\frac{{N_{AAC} }}{{N_{total} }}, \ldots ,\frac{{N_{TTG} }}{{N_{total} }},\frac{{N_{TTT} }}{{N_{total} }}} \right)_{k = 3}$$

7 $$TeNC = \left( {\frac{{N_{AAAA} }}{{N_{total} }},\frac{{N_{AAAC} }}{{N_{total} }}, \ldots ,\frac{{N_{TTTG} }}{{N_{total} }},\frac{{N_{TTTT} }}{{N_{total} }} } \right)_{k = 4}$$

where $N_{Kmer}$ donates the number of a certain nucleotide composition $Kmer$, and $k$ is the sequence length of nucleotide composition. That is, when $k = 1$, $Kmer$ only contains one base $p$; when $k = 2$, $Kmer$ consists of dinucleotide pairs $p$, $q$ constitutes; and so on, we set $1 \le k \le 4$ here. $N_{total}$ represents the sum of *k*-tuple nucleotide composition from the given sequence. NAC, DNC, TNC, and TeNC will produce 4-, 16-, 64- and 256-dimensional feature vectors. After splicing, the gene sequence will be encoded into 340-dimensional feature vectors.

*DPCP* We used the following 21 dinucleotide physicochemical properties for the computation of feature encoding DPCP, including Base stacking, Protein induced deformability, B-DNA twist; A-philicity, Propeller twist, Duplex stability (free energy), Duplex stability (disrupt energy), DNA denaturation, Bending stiffness, Protein DNA twist, Stabilizing energy of Z-DNA, Aida_BA_transition, Breslauer_dG, Breslauer_dH, Breslauer_dS, Electron_interaction, Hartman_trans_free_energy, Helix-Coil_transition, Ivanov_BA_transition, Lisser_BZ_transition and Polar_interaction. The calculation of this feature vector can be expressed as:

8 $$D_{DPCP} = \left[ {pc_{1} \left( {AA} \right)*f_{AA} , \ldots ,pc_{1} \left( {TT} \right)*f_{TT} , \ldots ,pc_{n} \left( {TT} \right)*f_{TT} } \right]^{T}$$

9 $$f_{XY} = \frac{{N_{XY} }}{{N_{total} }}$$

where $XY$ denotes dinucleotides, and $pc_{n} \left( {XY} \right)$ is the value of the dinucleotide $XY$ corresponding to the physicochemical property of the nth dinucleotide. $f_{XY}$ is the ratio of the number of dinucleotides $XY$, namely $N_{XY}$, to the total number of dinucleotides, namely $N_{total}$. After DPCP encoding, we finally get a dimension feature vector of 336 ($16 \times 21{ }$).

*TPCP* In this study, we considered 10 trinucleotide physicochemical properties: Bendability-DNAse, Bendability-consensus, Trinucleotide GC Content, Nucleosome positioning, Consensus_roll, Dnase I, Dnase I-Rigid, MW-Daltons, Nucleosome and Nucleosome-Rigid. The calculation method of TPCP is similar to DPCP, and its feature vector representation is as follows:

10 $$D_{TPCP} = \left[ {pc_{1} \left( {AAA} \right)*f_{AAA} , \ldots ,pc_{1} \left( {TTT} \right)*f_{TTT} , \ldots ,pc_{n} \left( {TTT} \right)*f_{TTT} } \right]^{T}$$

11 $$f_{XYZ} = \frac{{N_{XYZ} }}{{N_{total} }}$$

where $XYZ$ refers to trinucleotides, $N_{XYZ}$ refers to the number of trinucleotides $XYZ$, and $N_{total}$ is the sum of the numbers of all trinucleotides. $pc_{n} \left( {XYZ} \right)$ is the value of ternary nucleotide $XYZ$ corresponding to the physicochemical properties of the $n$ th trinucleotide. $f_{XYZ}$ represents the frequency of occurrence of trinucleotide $XYZ$ in a given gene sequence, that is, the proportion of the total number of trinucleotides. Finally, TPCP is encoded as a dimensional feature vector of 640 ($64 \times 10$).

*EIIP* Nair et al. calculated the energy of delocalized electrons in nucleotides as electron–ion interaction pseudopotential, namely EIIP. The EIIP values corresponding to the four bases are {A: 0.1260, C: 0.1340, G: 0.0806, T: 0.1335}, respectively. We considered the EIIP encoding of trinucleotides, calculated as follows:

12 $$D_{EIIP} = \left[ {EIIP_{AAA} *f_{AAA} ,EIIP_{AAC} *f_{AAC} , \ldots ,EIIP_{TTT} *f_{TTT} } \right]^{T}$$

where $EIIP_{XYZ} = EIIP_{X} + EIIP_{Y} + EIIP_{Z}$, $XYZ$ represents the trinucleotide, and the EIIP value of the trinucleotide $XYZ$ is the sum of the EIIP values of the nucleotides $X$, $Y$, and $Z$. $f_{XYZ}$ has the same meaning as in TPCP, indicating the frequency of occurrence of trinucleotide $XYZ$. The final EIIP provides a 64-dimensional feature vector.

*PseKNC* is the pseudo k-tuple nucleotide composition widely adopted in researchers' methods due to its powerful information extraction ability for DNA and RNA sequences, such as iTerm-PseKNC [43]. PseKNC knows how to utilize multiple physicochemical properties to cover a large amount of local and global sequence order information in feature vectors. Meanwhile, it possesses two variants: type I PseKNC and type II PseKNC. Here we use type II PseKNC, which is defined as follows:

13 $$D_{PseKNC}^{II} = \left[ {d_{1} , \ldots ,d_{{4^{K} }} ,d_{{4^{K} + 1}} , \ldots ,d_{{4^{K} + \lambda }} ,d_{{4^{K} + \lambda + 1}} , \ldots ,d_{{4^{K} + \lambda \Lambda }} } \right]^{T}$$

14 $$d_{u} = \left\{ {\begin{array}{*{20}l} {\frac{{f_{u}^{K - tuple} }}{{\mathop \sum \nolimits_{i = 1}^{{4^{K} }} f_{i}^{K - tuple} + \omega \mathop \sum \nolimits_{j = 1}^{{\lambda {\Lambda }}} \tau_{j} { }}}} \hfill & {\left( {1 \le \mu \le 4^{K} } \right)} \hfill \\ {\frac{{\omega \tau_{{\mu - 4^{K} }} }}{{\mathop \sum \nolimits_{i = 1}^{{4^{K} }} f_{i}^{K - tuple} + \omega \mathop \sum \nolimits_{j = 1}^{{\lambda {\Lambda }}} \tau_{j} { }}}} \hfill & {\left( {4^{K + 1} \le \mu \le 4^{K} + \lambda {\Lambda }} \right)} \hfill \\ \end{array} } \right.$$

where $L$ is the length of a given gene sequence, and $K$ represents a $K$-tuple nucleotide composition. ${\Lambda }$ is the number of physicochemical properties. $\lambda$ reflects the rank or level of correlation along the DNA sequence, and $\lambda$ is an integer less than $L - k$. $\omega$ is a weight factor. $f_{u}^{K - tuple}$ is the normalized occurrence frequency of the $\mu$ th $K$-tuple nucleotide in the DNA sequence. $\tau_{j}$ is the $j$-tier correlation factor, which is defined as follows:

15 $$\left\{ {\begin{array}{*{20}l} {\tau_{1} = \frac{1}{L - K - 1}\mathop \sum \limits_{i = 1}^{L - K - 1} J_{i,i + 1}^{1} } \hfill \\ { \tau_{2} = \frac{1}{L - K - 1}\mathop \sum \limits_{i = 1}^{L - K - 1} J_{i,i + 1}^{2} } \hfill \\ { \ldots \ldots \ldots \ldots } \hfill \\ {\tau_{{\Lambda }} = \frac{1}{L - K - 1}\mathop \sum \limits_{i = 1}^{L - K - 1} J_{i,i + 1}^{{\Lambda }} \quad \lambda < \left( {L - k} \right)} \hfill \\ { \ldots \ldots \ldots \ldots } \hfill \\ {\tau_{{\lambda {\Lambda } - 1}} = \frac{1}{L - K - \lambda }\mathop \sum \limits_{i = 1}^{L - K - \lambda } J_{i,i + \lambda }^{{{\Lambda } - 1}} } \hfill \\ {\tau_{{\lambda {\Lambda }}} = \frac{1}{L - K - \lambda }\mathop \sum \limits_{i = 1}^{L - K - \lambda } J_{i,i + \lambda }^{{\Lambda }} } \hfill \\ \end{array} } \right.$$

in which

16 $$\left\{ {\begin{array}{*{20}l} {J_{i,i + m}^{\xi } = {\rm H}_{\xi } \left( {R_{i} R_{i + 1} \ldots R_{i + K - 1} } \right) \cdot {\rm H}_{\xi } \left( {R_{i + m} R_{i + m + 1} \ldots R_{i + m + K - 1} } \right)} \hfill \\ {\xi = 1,2, \ldots ,\Lambda ; \quad m = 1,2, \ldots ,\lambda ; \quad i = 1,2, \ldots ,L - K - \lambda } \hfill \\ \end{array} } \right.$$

where ${\rm H}_{\xi } \left( {R_{i} R_{i + 1} \ldots R_{i + K - 1} } \right)$ and ${\rm H}_{\xi } \left( {R_{i + m} R_{i + m + 1} \ldots R_{i + m + K - 1} } \right)$ is the normalized value of the $\xi {\text{th}}$ physicochemical property of *K*-tuple nucleotides $R_{i} R_{i + 1} \ldots R_{i + K - 1}$ and $R_{i + m} R_{i + m + 1} \ldots R_{i + m + K - 1}$ in the DNA sequence.

Type II PseKNC encoding finally produces a ($4^{K} + \lambda \Lambda$) dimension feature vector. Here, we first tried different $K,\lambda ,n,\omega$ as well as PseDNC (pseudo dinucleotide composition) and PseTNC (pseudo trinucleotide composition) in the cell line GM12878, and tested the effect using LightGBM for a suitable set of hyperparameters. Then, we set $K,\lambda ,n,\omega$ to 4, 5, 2, 0.1, respectively, and used 6 physicochemical properties of dinucleotide (Twist, Tilt, Roll, Shift, Slide, Rise). Finally, the procedure was produced a feature vector of 286 ($4^{4} + 5 \times 6$) dimension.

### Conventional classifiers

**Fig. 5:**
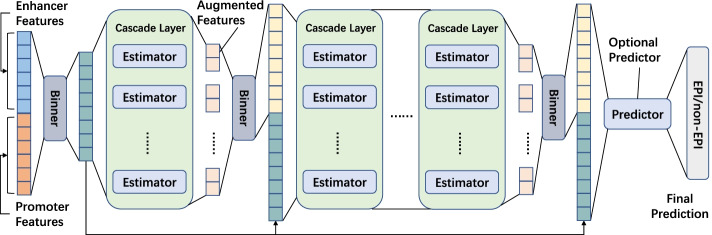
DF model structure. It consists of four main components: Binner, Cascade Layer, Estimator, and Predictor. Binner is used to reduce feature input, Estimator is used to form cascade layers, and Cascade Layer is used to process layer by layer and generate the next layer of feature information. Predictor is an estimator for the final prediction result, which is optional

*DF* was originally proposed by Zhihua Zhou et al. in 2017, which is an ensemble method of decision trees. Compared with deep learning networks, it has the advantages of fewer hyperparameter settings and automatic determination of model complexity dependent on data. The core of DF is the multi-grained cascade forest (gcForest) method, which includes two important parts: cascade forest structure and multi-grained scanning. The model structure is presented in Fig. 5. We obtained the package deep-forest following the instructions at https://github.com/LAMDA-NJU/Deep-Forest and used the CascadeForestClassifier classifier for it this study.

*SVM* is a traditional supervised machine learning method based on the structural risk minimization (SRM) [44] framework introduced in the late 1990s. The basic principle of SVM is to map the initial feature vector to a higher-dimensional Hilbert space and find an optimal separation hyperplane in the feature space to maximize the interval between positive and negative samples. SVM also uses kernel functions to deal with nonlinear classification problems [45]. Commonly used kernel functions are linear, polynomial, and Gaussian radial basis (RBF). We find that the Gaussian RBF kernel is the most suitable for this problem. In this study, to improve the training efficiency, we adopt the python package thundersvm [46] that can run on GPU, available from https://github.com/Xtra-Computing/thundersvm.

*LightGBM* is another efficient implementation of gradient boosting decision tree (GBDT) [47] algorithm besides XGBoost and pGBRT [48]. LightGBM holds the characteristics of higher efficiency, faster training speed, and lower memory consumption, and LightGBM can support distributed and rapid processing of large amounts of data. Two different technologies are mainly adopted in LightGBM: gradient-based one-side sampling (GOSS) and exclusive feature bundling (EFB). LightGBM uses GOSS to exclude data instances with small gradients and retain only those data instances with larger gradients that play a more significant role in calculating information gain while exploiting EFB to bundle mutually exclusive to reduce the number of features. We used the LightGBM package that obtained the python version provided by Microsoft from https://github.com/microsoft/Light-GBM/tree/master/python-package.

*XGBoost* is a scalable machine learning system implemented by Chen and Guestrin in 2016 based on the gradient boosting framework. First of all, in principle, XGBoost introduces regularization to prevent overfitting of the trained model and uses an approximate algorithm to solve the problem that the greedy algorithm cannot make data read into memory for calculation when the amount of data is too large. Secondly, XGBoost relies on weighted quantile thumbnails to process weighted data to propose candidate split points and then applies the sparsity aware algorithm to deal with sparse data and missing values. Finally, XGBoost adopts fast structure design, out-of-core computing, and cache-aware learning in system design. Therefore, XGBoost has the advantages of supporting distributed and parallel computing and fast, massive data processing. The python version of the XGBoost package is delivered by https://github.com/dmlc/xgboost/tree/master/python-package.

*RF* is one of the most widely used maximum likelihood algorithms. The core idea of RF is to integrate multiple independent and unrelated decision trees in the form of bagging integration and introduce randomness (bootstrap sample method and random selection of feature subsets) to prevent the risk of model overfitting and improve anti-noise ability. The following is the construction process of the decision tree:

1. For the training set with $N$ samples, the bootstrap sample method is used to extract $N$ samples from it as the training set of the decision tree;
2. Assuming that the number of features is $M$, and $m \left( {m \ll M} \right)$ features are randomly selected to form a feature subset. When each node of the decision tree needs to be split, the optimal feature is selected from the feature subset as the division attribute of the node;
3. Each tree grows as much as possible without pruning.

*MLP* is an artificial neural network that structurally includes input, hidden, and output layers, uses backpropagation to update weight computationally and is used to solve nonlinear problems. It has been widely used in problems in different fields.

*LR* is a maximum likelihood algorithm for solving classification problems, and it has been successfully applied to many classification tasks in bioinformatics [49, 50].

### Stacking structure

Our study proposes a stacking ensemble learning framework, StackEPI, which can precisely identify cell-specific EPIs from only genetic sequence data. An overview of StackEPI is illustrated in Fig. 1, including data preprocessing (in Fig. 1a), feature extraction (in Fig. 1b), and integrated framework (in Fig. 1c). The detailed description of the data preprocessing and feature extraction is given above, and the model structure is emphasized here. StackEPI uses 5 outperforming machine learning algorithms and 6 sequence-based feature encodings to build a total of 30 baseline models (i.e. first-level). Then we utilize the predicted probability information obtained from these baseline models as a new feature input into the MLP to obtain the MLP meta-model (i.e. second-level). Finally, we use the predictions we get from the meta-model as the final predictions.

### Model training setup

As mentioned above, our dataset is an unbalanced dataset with a positive-to-negative sample ratio of 1:20. In supervised learning, the classification model always tends to focus on the majority class and reduce the prediction accuracy of the minority class. Therefore, we perform data augmentation operations on the training set and test the performance of our model on the independent test set. For a fair comparison with EPIVAN and EPI-DLMH, the specific training process of the ensemble model for any given cell line is described as follows:

1. The original unbalanced dataset $D$ is randomly divided into a training set $D_{train}$ (90% of $D$) and independent test set $D_{test}$ (10% of $D$) by stratified sampling;
2. Augment the training set $D_{train}$ to produce a balanced training set $D_{aug}$;
3. Train the model on the balanced training set $D_{aug}$;
4. Evaluate the model on the independent test set $D_{test}$.

To make the ensemble model achieve better performance, we use grid search to tune the essential parameters of the base classifiers and meta-classifiers of the ensemble model. If only a one-time grid search adjusts all parameters for some classifiers that need to be tuned with many parameters, it will result in a considerable time cost. Therefore, we group the parameters of such classifiers and then sequentially adjust them layer by layer. The optimized machine learning parameters and search ranges are given in Additional file 1: Table S1.

### Performance evaluation

This study uses AUROC, AUPR, and F1-score to evaluate our method. The receiver operating characteristic curve reflects the relationship between sensitivity and specificity at different thresholds. The precision-recall curve reflects the tradeoff between the model's accuracy for identifying positive examples and the model's ability to cover positive examples. The closer the values of AUROC, AUPR, and F1-score are to 1, the better the model's performance.

## Supplementary Information

**Additional file 1.** Parameters and the value range of parameter adjustment.

## Data Availability

The datasets supporting the conclusions of this article are included with article. Project name: StackEPI. Project home page: https://github.com/20032303092/StackEPI.git. Project inclusion: All datasets and the code needed to replicate the experiment.

## Abbreviations

3C: Chromosome conformation capture
AUROC: Area under receiver operating characteristic curve
AUPR: Area under precision-recall curve
ChIA-PET: Chromatin interaction analysis by paired-end tag sequencing
CNN: Convolutional neural network
DF: Deep Forest
DNC: Dinucleotide composition
EFB: Exclusive feature bundling
EPIs: Enhancer–promoter interactions
GBDT: Gradient boosting decision tree
gcForest: Multi-grained cascade forest
GOSS: Gradient-based one-side sampling
Hi-C: High-throughput chromosome conformation capture
LR: Logistic Regression
LSTM: Long short-term memory
MLP: Multi-Layer Perceptron
NAC: Nucleotide acid composition
PseDNC: Pseudo dinucleotide composition
PseTNC: Pseudo trinucleotide composition
RBF: Radial basis function
RF: Random Forest
SVM: Support Vector Machine
TeNC: Tetranucleotide composition
TNC: Trinucleotide composition
