# Supplementary material for: StackEPI: identification of cell line-specific enhancer–promoter interactions based on stacking ensemble learning
Source: BMC Bioinformatics. 2022 Jul 11;23:272. doi: 10.1186/s12859-022-04821-9 (PMC9277947; doi:10.1186/s12859-022-04821-9)
Supplement: Supplementary file 1 — Additional file 1. Parameters and the value range of parameter adjustment. [file 12859_2022_4821_MOESM1_ESM.docx]

**Table S1** Parameters and the value range of parameter adjustment

| **Classifier** | **Parameter** | **Value** |
| --- | --- | --- |
| DF | n_estimators, n_trees, max_layers | [2, 5, 8, 10, 13], [50, 400] Δ = 50, [10, 25] Δ = 5 |
| LightGBM | {max_depth, num_leaves}, {max_bin, min_child_samples}, {colsample_bytree, subsample, subsample_freq}, {reg_alpha, reg_lambda}, {min_split_gain}, {learning_rate, n_estimators} | {[-1, 13] Δ = 1, [221, 350] Δ = 10}, {[5, 256] Δ = 10, [10, 201] Δ = 10}, {[0.6, 1.0] Δ = 0.1, [0.6, 1.0] Δ = 0.1, [0, 81] Δ = 10}, {[1e-5, 1e-3, 1e-1, 0.0, 0.1, 0.3, 0.5, 0.7, 0.9, 1.0], [1e-5, 1e-3, 1e-1, 0.0, 0.1, 0.3, 0.5, 0.7, 0.9, 1.0]}, {[0.0, 1.0] Δ = 0.1}, {[0.001, 0.01, 0.05, 0.07, 0.1, 0.2, 0.5, 0.75, 1.0], [50, 250] Δ = 25} |
| RF | {n_estimators}, {max_depth}, {min_samples_split, min_samples_leaf}, {max_features} | {[10, 350] Δ = 10}, {[1, 150] Δ = 1}, {[2, 10] Δ = 1, [1, 10] Δ = 1}, {[auto, sqrt, log2, None]} |
| SVM | C, gamma | [$2^{-4}$, $2^{11}$] Δ = 2, [$2^{-4}$, $2^{12}$] Δ = 2 |
| XGBoost | {n_estimators}, {max_depth, min_child_weight}, {gamma}, {subsample, colsample_bytree}, {reg_alpha, reg_lambda}, {learning_rate} | {[50,100] Δ = 50}, {[3,12] Δ = 1, [1,6] Δ = 1}, {[0, 0.6] Δ = 1}, {[0.6,0.9] Δ = 0.1, [0.6,0.9] Δ = 0.1}, {[0, 0.01, 0.02, 0.05, 0.1, 0.5, 1, 2, 3], [0, 0.01, 0.02, 0.05, 0.1, 0.5, 1, 2, 3]}, {[0.001, 0.01, 0.05, 0.07, 0.1, 0.2, 0.5, 0.75, 1.0]} |
| LR | C | [0.1, 3] Δ = 0.2 |
| MLP | {batch_size, learning_rate_init, solver, max_iter}, {activation, hidden_layer_sizes}, {alpha} | {[64, 128], [0.0001, 5e-6], [adam, sgd, lbfgs], 300}, {[identity, logistic, tanh, relu], [8, 32, (16, 32), (8, 16)]}, {[1e-5, 1e-3, 1e-1, 0.0, 0.1, 0.3, 0.5, 0.7, 0.9, 1.0]} |

Δ represents the step size, {} represents grouping parameters.
